# Inflammatory and Nutritional Markers Predicting Pathological Complete Response to Neoadjuvant Therapy in HER2-Positive Breast Cancer: A Multicenter Real-World Study

**DOI:** 10.3390/jcm14207271

**Published:** 2025-10-15

**Authors:** Zeliha Birsin, İsmail Nazlı, Onur Alkan, Hülya Odabaşı Bükün, Murat Günaltılı, Emir Çerme, Vali Aliyev, Selin Cebeci, Seda Jeral, Hamza Abbasov, Türkkan Evrensel, Çiğdem Papila, Berrin Papila, Ceyda Sönmez Wetherilt, Nebi Serkan Demirci, Özkan Alan

**Affiliations:** 1 Department of Medical Oncology, Cerrahpaşa Faculty of Medicine, Istanbul University–Cerrahpaşa, Istanbul 34320, Türkiye; murat.gunaltili@iuc.edu.tr (M.G.); emircrm34@gmail.com (E.Ç.); dktr.aliyev@gmail.com (V.A.); sellcebeci@gmail.com (S.C.); sedajeral@gmail.com (S.J.); hamzaabbasov90@gmail.com (H.A.); bpapila@iuc.edu.tr (Ç.P.); drserkannebi@yahoo.com (N.S.D.); ozkan.alan@hotmail.com (Ö.A.); 2 Department of Medical Oncology, Göztepe Prof. Dr. Süleyman Yalçın City Hospital, Istanbul 34722, Türkiye; mdi.nazli@gmail.com (İ.N.); dronuralkan@gmail.com (O.A.); 3 Department of Medical Oncology, Faculty of Medicine, Bursa Uludağ University, Bursa 16059, Türkiye; hulyaodabasi@uludag.edu.tr (H.O.B.); evrensel@uludag.edu.tr (T.E.); 4 Department of General Surgery, Cerrahpaşa Faculty of Medicine, Istanbul University–Cerrahpaşa, Istanbul 34320, Türkiye; berrin.papila@iuc.edu.tr; 5 Department of Pathology, Cerrahpaşa Faculty of Medicine, Istanbul University–Cerrahpaşa, Istanbul 34320, Türkiye; ceyda.wetherilt@iuc.edu.tr

**Keywords:** HER2-positive breast cancer, pathological complete response (pCR), neoadjuvant therapy (NAT), prognostic nutritional index (PNI), systemic inflammatory markers, neutrophil-to-lymphocyte ratio (NLR), platelet-to-lymphocyte ratio (PLR), lymphocyte-to-monocyte ratio (LMR)

## Abstract

**Background:** Pathological complete response (pCR) following neoadjuvant therapy (NAT) is a key surrogate marker for long-term outcomes in HER2-positive breast cancer. Identifying clinical and biological predictors of pCR, including systemic inflammatory and nutritional markers such as the neutrophil-to-lymphocyte ratio (NLR), platelet-to-lymphocyte ratio (PLR), lymphocyte-to-monocyte ratio (LMR), neutrophil-to-albumin ratio (NAR), C-reactive protein-to-albumin ratio (CAR), systemic immune-inflammation index (SII), and prognostic nutritional index (PNI), may help refine treatment strategies and improve patient outcomes. **Methods:** We retrospectively analyzed 174 patients with stage II–III HER2-positive breast cancer who received neoadjuvant anti-HER2-based regimens at multiple centers between 2010 and 2025. Demographic, clinicopathological, and laboratory data were collected, and inflammatory and nutritional indices (NLR, PLR, LMR, NAR, CAR, SII, PNI) were calculated. Predictors of pCR were evaluated using univariate and multivariate logistic regression analyses. **Results:** Overall, 49% of patients achieved pCR. In multivariate analysis, independent predictors of pCR were hormone receptor negativity, smaller tumor size, HER2 IHC 3+ expression, dual HER2 blockade, and a higher prognostic nutritional index (PNI ≥ 55). In contrast, systemic inflammatory indices such as NLR, PLR, LMR, NAR, CAR, and SII were not significantly associated with pCR. **Conclusions:** This multicenter real-world study demonstrates that conventional inflammatory markers have limited predictive value, whereas the PNI emerges as a simple and practical biomarker reflecting nutritional and immune status. Integrating PNI with clinicopathological factors may enhance risk stratification and help guide individualized neoadjuvant treatment strategies in HER2-positive breast cancer.

## 1. Introduction

Breast cancer (BC) is a biologically heterogeneous disease characterized by distinct molecular subtypes, each demonstrating variable clinical behaviors and therapeutic responses [1]. Among these, human epidermal growth factor receptor 2 (HER2)-positive breast cancer accounts for approximately 15–20% of all cases [2].

Neoadjuvant HER2-targeted therapy has become the standard initial treatment approach for HER2-positive breast cancer [2,3]. Achieving a pathological complete response (pCR) after neoadjuvant therapy (NAT) is considered a surrogate marker for improved long-term outcomes [4]. However, substantial heterogeneity in tumor biology, immune status, and therapeutic sensitivity is reflected in the markedly variable pCR rates observed among patients with HER2-positive disease [3].

Systemic inflammation is known to play a key role in promoting tumor progression, invasion, and metastasis [5]. Therefore, in recent years, inflammation-based and immune-nutritional biomarkers have gained attention for their potential to predict oncologic outcomes and treatment response [6,7,8,9,10,11,12]. As indicators of systemic inflammation, the neutrophil-to-lymphocyte ratio (NLR), lymphocyte-to-monocyte ratio (LMR), platelet-to-lymphocyte ratio (PLR) and the systemic immune-inflammation index (SII), are frequently researched indices [13]. Additional metrics that offer information on nutritional and immuno-inflammatory status include the prognostic nutritional index (PNI), the C-reactive protein-to-albumin ratio (CAR), the neutrophil-to-albumin ratio (NAR) [11,12]. Numerous studies have explored the prognostic and predictive value of these markers in breast cancer; however, the evidence remains mixed.

Our study is a real-life, multicenter analysis that investigates the relationship between inflammatory and nutritional biomarkers and pathological complete response in patients with HER2-positive breast cancer undergoing neoadjuvant HER2-targeted therapy. Importantly, both laboratory and clinical parameters were evaluated together to provide a more comprehensive and integrated assessment of predictors of treatment response in this specific patient population.

## 2. Materials and Methods

### 2.1. Study Design and Patient Inclusion Criteria

This retrospective, multicenter study included patients diagnosed with stage II–III HER2-positive breast cancer between July 2010 and January 2025. Data were collected from three medical oncology centers in Türkiye. Eligible patients were aged 18 years or older and had confirmed HER2-positive status, defined as immunohistochemistry (IHC) 3+ or IHC 2+ with fluorescence in situ hybridization (FISH) positivity. Only patients who had completed neoadjuvant anti-HER2-based therapy (either monotherapy or dual blockade) and subsequently underwent surgery were included in the study.

Patients were excluded if they met any of the following criteria: age under 18 years, presence of metastatic disease at diagnosis, failure to initiate or complete NAT, progression during treatment, inability to undergo surgery, diagnosis of a second primary malignancy, ECOG performance status greater than 2, the presence of a known hematologic disorder, autoimmune disease, bilateral invasive breast cancer with differing molecular subtypes, or incomplete clinical or pathological data.

### 2.2. Data Collection, Study Variables and Outcome Definitions

Archived patient files and institutional electronic medical records were reviewed to obtain clinical, pathological, and treatment-related data. Data were retrospectively collected from three oncology centers using standardized data collection templates that included demographic, clinical, pathological, and laboratory parameters. All centers used comparable laboratory reference ranges, and where minor discrepancies were identified, results were standardized to a common scale prior to analysis. To minimize inter-center variability, the dataset was independently reviewed and validated by two investigators before statistical analysis. Collected data included demographic characteristics (age, sex) and clinical features (comorbidities, ECOG performance status, body mass index [BMI]). Tumor-related variables included tumor laterality (right, left, or bilateral), histological type and grade, estrogen receptor (ER), progesterone receptor (PR), Ki-67 proliferation index, and HER2 expression status (defined as IHC 3+ or IHC 2+ with FISH-confirmed amplification). Hormone receptor positivity was defined as >1% expression of ER or PR on immunohistochemical staining from diagnostic biopsy samples.

Clinical tumor (cT) and nodal (cN) stages at diagnosis were recorded and staging was performed according to the American Joint Committee on Cancer (AJCC) Staging Manual, 8th edition. Treatment-related data included whether the patient received an anthracycline-based chemotherapy regimen and whether dual anti-HER2 blockade was administered during neoadjuvant therapy. The date of surgery following completion of neoadjuvant therapy was also documented.

In addition, baseline laboratory values including complete blood counts and routine biochemistry results were collected prior to the initiation of neoadjuvant treatment. Inflammatory and nutritional markers such as the NLR, PLR, LMR, CAR, PNI, and SII were calculated using these baseline values.

pCR, which is the absence of residual invasive breast cancer in both the breast and axillary lymph nodes (ypT0/is, ypN0), was the main outcome of interest. This definition was used as the standard criterion for pCR assessment in the present study.

Inflammatory and Nutritional Indices:

NLR (Neutrophil-to-Lymphocyte Ratio) = Neutrophil count (10^3^/mm^3^)/Lymphocyte count (10^3^/mm^3^).

PLR (Platelet-to-Lymphocyte Ratio) = Platelet count (10^3^/µL)/Lymphocyte count (10^3^/mm^3^).

LMR (Lymphocyte-to-Monocyte Ratio) = Lymphocyte count (10^3^/mm^3^)/Monocyte count (10^3^/mm^3^).

PNI (Prognostic Nutritional Index) = Serum albumin (g/L) + 5 × Lymphocyte count (10^3^/mm^3^).

SII (Systemic Immune-Inflammation Index) = Platelet count (10^3^/µL) × Neutrophil count (10^3^/mm^3^)/Lymphocyte count (10^3^/mm^3^).

CAR (C-reactive Protein-to-Albumin Ratio) = CRP (mg/L)/Serum albumin (g/L).

NAR (Neutrophil-to-Albumin Ratio) = Neutrophil count (10^3^/mm^3^)/Serum albumin (g/L).

### 2.3. Statistical Analysis

All statistical analyses were conducted using IBM SPSS Statistics software, version 26.0 (IBM Corp., Armonk, NY, USA). The distribution of continuous variables was evaluated using both the Shapiro–Wilk and Kolmogorov–Smirnov tests, supported by visual assessments such as Q–Q plots and histograms. Continuous variables were expressed as mean ± standard deviation for normally distributed data or as median and range for non-normally distributed data. Categorical variables were summarized using frequencies and percentages. Receiver operating characteristic (ROC) curve analysis was used to determine optimal cut-off values for inflammatory and nutritional markers.

Group comparisons were made using the independent samples *t*-test for normally distributed continuous variables, and the Mann–Whitney U test for non-normally distributed data. For categorical variables, the Chi-square test or Fisher’s exact test was used as appropriate.

To identify independent predictors of pCR, univariate and multivariate logistic regression analyses were conducted. Variables found to be statistically significant in univariate analysis or considered clinically relevant were included in the multivariate model. A two-sided *p*-value < 0.05 was considered statistically significant in all analyses.

### 2.4. Ethical Considerations

This study was conducted in accordance with the principles outlined in the Declaration of Helsinki and adhered to all applicable institutional and international ethical guidelines. Access to patient data was restricted to the physicians responsible for data analysis and manuscript preparation, in line with institutional confidentiality regulations. Ethical approval for the study was obtained from the Institutional Review Board (IRB) of Istanbul University–Cerrahpaşa, Cerrahpaşa Faculty of Medicine (Approval No: 508/2025, dated 13 July 2025).

## 3. Results

### 3.1. Baseline Clinical, Demographic, and Treatment Characteristics

A total of 174 patients with stage II–III HER2-positive breast cancer who received neoadjuvant anti-HER2-based therapy and subsequently underwent surgery were included in the study. Of these, 86 patients (49%) achieved a pCR, whereas 88 (51%) did not.

The median age of the entire cohort was 51 years (min 25–max 80), and no significant difference was observed between the pCR and non-pCR groups regarding age distribution, comorbidities, BMI, menopausal status, tumor laterality, or histological subtype (*p* > 0.05 for all).

While patients with grade 3 tumors had a higher rate of pCR compared to those with grade 2, the difference did not reach statistical significance (*p* = 0.062). Similarly, clinical T1–2 tumors were more common overall, and although not statistically significant (*p* = 0.055). Stage II disease was more common than stage III (68% vs. 32%), with a trend toward higher pCR in stage II (*p* = 0.065). The majority had N0–N1 disease (87%), nodal status was not associated with pCR (*p* = 0.540).

Hormone receptor status was significantly correlated with pCR, with hormone receptor-negative tumors achieving higher response rates compared to hormone receptor-positive tumors (45% vs. 28%, *p* = 0.021). Likewise, HER2 IHC 3+ positivity was also significantly associated with pCR compared to IHC 2 +/SISH+ cases (94% vs. 6%, *p* = 0.004).

Patients who received dual anti-HER2 therapy had significantly higher pCR rates than those who received monotherapy (83% vs. 67%, *p* = 0.019). Anthracycline-based chemotherapy was administered to 83% of the cohort, but there was no significant difference in pCR rates based on whether anthracyclines were used (*p* = 0.740).

At the time of data collection, 91% of the patients were still alive. Recurrence rates were significantly lower in the pCR group compared with the non-pCR groups (8% vs. 19%, *p* = 0.033). All these findings are summarized in Table 1.

### 3.2. Inflammatory and Nutritional Markers in Relation to pCR

ROC curve analysis was initially performed to determine optimal cut-off values for the inflammatory and nutritional indices in predicting pCR. However, none of the markers yielded statistically significant area under the curve (AUC) values that could define clinically meaningful thresholds (Table S1). Due to the absence of validated cut-off points, each marker was dichotomized based on its median value within the study cohort for subsequent categorical analysis.

The relationship between pCR status and inflammatory and nutritional indices was assessed. No statistically significant association was found between pCR and NLR (*p* = 0.361), PLR (*p* = 0.881), LMR (*p* = 0.068), SII (*p* = 0.363), CAR (*p* = 0.765), or NAR (*p* = 0.580). Among all indices, only PNI showed a statistically significant difference: patients with PNI ≥ 55 had a higher rate of pCR compared to those with PNI < 55 (71% vs. 56%, *p* = 0.037) (Table 2)

### 3.3. Predictors of pCR

To investigate the factors associated with achieving a pCR, logistic regression analyses were performed. Initially, univariate logistic regression identified several variables with either statistical significance or borderline significance. These included hormone receptor status (*p* = 0.021), HER2 status (*p* = 0.007), tumor size (T stage, *p* = 0.058), clinical stage (stage II vs. III, *p* = 0.067), tumor grade (*p* = 0.062), dual anti-HER2 therapy (*p* = 0.02), and PNI (*p* = 0.038).

In multivariate logistic regression analysis, five factors remained independently associated with pCR. Hormone receptor negativity significantly increased the likelihood of pCR (OR: 2.59, 95% CI: 1.25–5.36, *p* = 0.01). Tumors with HER2 IHC 3+ expression were more likely to respond compared to IHC 2 +/SISH + tumors (OR: 0.23, 95% CI: 0.07–0.71, *p* = 0.011). Patients with smaller tumors (T1–2) had higher odds of achieving pCR (OR: 0.34, 95% CI: 0.14–0.79, *p* = 0.012). Receiving dual anti-HER2 therapy was also a significant independent predictor (OR: 2.49, 95% CI: 1.16–5.33, *p* = 0.019). Finally, a PNI ≥ 55 was strongly associated with better response (OR: 2.89, 95% CI: 1.40–5.99, *p* = 0.004). Findings are summarized in Table 3.

## 4. Discussion

In this multicenter, real-world study, we evaluated the clinical, pathological, and immune-nutritional factors associated with pCR in patients with stage II–III HER2-positive breast cancer treated with neoadjuvant anti-HER2-based therapy. By integrating routinely available laboratory markers with established clinicopathologic variables, we aimed to comprehensively assess predictors of treatment response in this specific population. Our results showed that obtaining pCR was independently correlated with hormone receptor negativity, HER2 IHC 3+ expression, T1–2 tumor stage, dual anti-HER2 blockade, and a higher PNI. Conversely, commonly studied systemic inflammatory markers such as NLR, PLR, LMR, SII, CAR, and NAR did not show significant predictive value for pCR, underscoring the complex and often inconsistent nature of their prognostic utility.

Several studies have investigated the prognostic value of hematological and biochemical serum markers in predicting breast cancer outcomes. In our study, inflammatory markers including NLR, PLR, LMR, NAR, CAR, and SII were evaluated. While previous reports suggested that lower NLR is associated with higher pCR rates, including in HER2-positive breast cancer [14,15], our analysis did not confirm these findings. Similarly, Corbeau et al. conducted a meta-analysis and reported no significant relationship between NLR and pCR [16]. With regard to PLR, findings in the literature are conflicting: while some studies, similar to ours, reported no association with response [17], others suggested that both higher and lower PLR levels might correlate with increased likelihood of achieving pCR after NAT [18,19]. In our cohort, a higher LMR demonstrated a borderline association with improved pCR rates in univariate analysis; however, this effect was not retained in the multivariate model. In the literature, both higher and lower LMR levels have been reported to correlate with pCR, reflecting inconsistency across studies [20,21]. Likewise, although higher NAR and CAR have been associated with better pCR in some reports [22,23], our data did not show any such relationship. Similarly, higher SII has been linked to lower pCR in the literature [24], but we did not observe this association in our study. These results suggest that the predictive role of systemic inflammatory markers in HER2-positive breast cancer remains uncertain.

In contrast, among all evaluated parameters, the PNI emerged as the only factor independently associated with pCR in our cohort, with patients having a PNI ≥ 55 showing a significantly higher likelihood of achieving pCR in multivariate analysis. This observation is consistent with the findings of Fanli Qu et al., who demonstrated in a large patient population that a PNI ≥ 53 was significantly associated with improved pCR rates [11]. Although we did not identify a significant ROC-based cutoff value in our analysis, the median PNI value used for subgrouping was comparable to those reported in previous studies [11,25,26].

The PNI, a composite score derived from serum albumin concentration and peripheral lymphocyte count, reflects both nutritional and immunological status [27]. Initially introduced to assess perioperative nutritional risk and predict postoperative complications in gastrointestinal malignancies [28], it has since been validated as a prognostic marker across a wide spectrum of cancers, including breast, renal cell carcinoma, pancreatic, and ovarian cancer [25,29,30,31]. Serum albumin serves as a readily available biomarker of protein reserves and systemic inflammation, while lymphocyte count reflects cell-mediated immunity, which is essential for antitumor defense [32,33]. Consequently, a reduced PNI indicates the dual burden of malnutrition and immunosuppression, both of which can negatively affect treatment response and survival. This condition may weaken the body’s immune surveillance, allowing the tumor to escape immune control and potentially promoting disease progression [34]. Patients with higher PNI values may be more likely to maintain adequate nutritional reserves and immune competence, leading to enhanced tolerance to chemotherapy and improved tumor response through better drug metabolism, tissue repair, and immune-mediated cytotoxicity [25]. In contrast, other systemic inflammatory indices such as NLR, PLR, and SII may be affected by transient conditions, including infections, stress, or comorbidities, which can limit their reliability as stable prognostic indicators [35,36].

In breast cancer, accumulating evidence highlights the prognostic significance of PNI. Two large meta-analyses demonstrated that higher PNI is associated with improved disease-free survival (DFS) and overall survival (OS) [25,26]. Moreover, higher PNI levels have been linked to increased pCR rates following neoadjuvant therapy in breast cancer [11,37], a finding consistent with the results of our study. In contrast to our results, Wang et al. reported that higher PNI was associated with worse pCR outcomes [38]. However, unlike other inflammation-based indices, PNI captures both immune competence and nutritional status, making it a biologically plausible and clinically relevant predictor.

The present study specifically focused on patients with stage II–III HER2-positive breast cancer treated with neoadjuvant therapy. In our cohort, hormone receptor (HR)-negative status was independently associated with higher pCR rates, smaller tumor size was also predictive of better response, and HER2 IHC 3+ tumors demonstrated significantly better response compared with IHC 2 +/FISH-positive cases, in line with previous reports [39,40,41]. Furthermore, dual HER2 blockade with trastuzumab plus pertuzumab yielded higher pCR rates compared with trastuzumab monotherapy, consistent with the existing literature [42]. Notably, among the evaluated clinical and pathological characteristics, PNI emerged as the only independent predictor of pCR among the evaluated inflammatory and nutritional scores, underscoring its potential clinical utility in guiding treatment strategies.

Our study also has some limitations. Its relatively small sample size and retrospective design may introduce selection bias and limits causal interpretation. Additionally, as only patients with an ECOG performance status < 2 were included, the study population was relatively healthy, which could limit the generalizability of the findings. The small sample size in certain subgroups (e.g., patients without anthracycline use or with HER2 IHC 2 +/SISH + status) may have reduced the power to detect differences in treatment response. Furthermore, because the cohort includes patients treated as early as 2010, a distinct subgroup received trastuzumab monotherapy as the sole anti-HER2 therapy, which could have influenced the observed outcomes (reflecting historical treatment standards).

## 5. Conclusions

This multicenter, real-world study provides additional insight into predictors of pathological complete response in patients with HER2-positive breast cancer receiving neoadjuvant anti-HER2 therapy. While traditional inflammatory indices showed limited predictive value, integration of clinical and biological parameters highlighted the importance of hormone receptor status, tumor size, HER2 expression level, dual blockade, and nutritional–immune status. Notably, the identification of PNI as an independent predictor emphasizes the need to consider host-related factors, not only tumor biology, when tailoring treatment strategies. Future prospective studies with larger and more homogeneous populations, including validation across different cohorts, are warranted to validate these findings and to further refine risk stratification in this setting.

## Figures and Tables

**Table 1:** Baseline clinicopathological characteristics of the overall cohort and comparison between patients with and without pCR.

Variables		Overall Cohort (*n*: 174)		Non-PCR (*n*: 88)		PCR (*n*: 86)		*p*
Age, median		51 (min 25–max 80)		51 (min 25–max 779		51 (min 29–max 80)		0.570 ^1^
Age, *n* (%)	<50	80	46%	40	45%	40	47%	0.889 ^2^
	≥50	94	54%	48	55%	46	53%	
Comorbidity, *n* (%)	Absent	105	60%	58	66%	47	55%	0.129 ^2^
	Present	69	40%	30	34%	39	45%	
BMI kg/m^2^, median		27.6 (min 17.9–max 42.9)		27.9 (min 17.9–max 42.9)		27.3 (min 18.2–max 42)		0.378 ^1^
BMI kg/m^2^, *n* (%)	<30	115	66%	57	65%	58	67%	0.710 ^2^
	≥30	59	34%	31	35%	28	33%	
Menopause status, *n* (%)	Premenopausal	73	42%	37	42%	36	42%	0.980 ^2^
	Postmenopausal	101	58%	51	58%	50	58%	
Primary Tumor Laterality, *n* (%)	Right	85	49%	40	45%	45	52%	0.365 ^2^
	Left	89	51%	48	55%	41	48%	
Histological Subtype, *n* (%)	IDC	152	87%	76	86%	76	88%	0.690 ^2^
	Other	22	13%	12	14%	10	12%	
Grade, *n* (%)	2	73	42%	43	49%	30	35%	0.062 ^2^
	3	101	58%	45	51%	56	65%	
Ki-67%, median		30 (min 5–max 95)		30 (min 5–max 95)		40 (min 5–max 90)		0.095 ^1^
Ki-67, *n* (%)	<30	71	41%	38	43%	33	38%	0.519 ^2^
	≥30	103	59%	50	57%	53	62%	
ER %, median		65 (min 0–max 100)		90 (min 0–max 100)		27 (min 0–max 100)		0.002 ^1^
PR %, median		0 (min 0–max 100)		0 (min 0–max 100)		0 (min 0–max 95)		0.240 ^1^
Subtype, *n* (%)	HR-negative	64	37%	25	28%	39	45%	0.021 ^2^
	HR-positive	110	63%	63	72%	47	55%	
Her2 status, *n* (%)	IHC 2+, FISH+	23	13%	18	20%	5	6%	0.004 ^2^
	IHC 3+	151	87%	70	80%	81	94%	
T stage, *n* (%)	T1–2	135	78%	63	72%	72	84%	0.055 ^2^
	T3–4	39	22%	25	28%	14	16%	
N stage, *n* (%)	N0–1	151	87%	75	85%	76	88%	0.540 ^2^
	N2–3	23	13%	13	15%	10	12%	
Stage, *n* (%)	2	118	68%	54	61%	64	74%	0.065 ^2^
	3	56	32%	34	39%	22	26%	
Neoadjuvant CT, *n* (%)	AC-THP	100	57%	43	49%	57	66%	0.038 ^2^
	AC-TH	44	25%	29	33%	15	17%	
	TCHP	30	17%	16	18%	14	16%	
Dual anti-Her2 therapy, *n (*%)	Not received	44	25%	29	33%	15	17%	0.019 ^2^
	Received	130	75%	59	67%	71	83%	
Anthracycline-based chemotherapy, *n* (%)	Not received	30	17%	16	18%	14	16%	0.740 ^2^
	Received	144	83%	72	82%	72	84%	
Recurrence, *n* (%)	Absent	150	86%	71	81%	79	92%	0.033 ^2^
	Present	24	14%	17	19%	7	8%	
Current status, *n* (%)	Alive	158	91%	78	89%	80	93%	0.317 ^2^
	Exitus	16	9%	10	11%	6	7%	

^1^ Mann–Whitney U test; ^2^ Chi-squared test; Abbreviations: pCR: Pathological Complete Response; BMI: Body Mass Index; IDC: Invasive Ductal Carcinoma; ER: Estrogen Receptor; PR: Progesterone Receptor; IHC: Immunohistochemistry; FISH: Fluorescence In Situ Hybridization; AC-THP: Adriamycin + Cyclophosphamide followed by Trastuzumab, Pertuzumab, and a Taxane; TH: Trastuzumab + Taxane; TCHP: Docetaxel, Carboplatin, Trastuzumab, and Pertuzumab.

**Table 2:** Inflammatory and nutritional markers in the overall cohort and their association with pCR.

Variables		Overall Cohort (*n*: 174)		Non-PCR (*n*: 88)		PCR (*n*: 86)		*p*
NLR, median		2.1 (min 0.4–max 12.2)		2.0 (min 0.4–max 7.3)		2.1(min 0.7–12.2)		0.350 ^1^
NLR, *n* (%)	<2.1	85	48%	46	52%	39	45%	0.361 ^2^
	≥2.1	89	52%	42	48%	47	55%	
PLR, median		133.1 (min 56–max 452)		133 (min 64–max 452)		134 (min 56–max 347)		0.520 ^1^
PLR, *n* (%)	<133	88	51%	45	51%	43	50%	0.881 ^2^
	≥133	86	49%	43	49%	43	50%	
LMR, median		4.2 (min 1.5–max 20)		4 (min 1.6–max 18)		4.4 (min 1.5–max 20)		0.180 ^1^
LMR, *n* (%)	<4.2	85	49%	49	56%	36	42%	0.068 ^2^
	≥4.2	89	51%	39	44%	50	58%	
NAR, median		0.9 (min 0.2–max 2.5)		0.9 (min 0.2–max 2.3)		0.8 (min 0.4–2.5)		0.816 ^1^
NAR, *n* (%)	<0.9	85	49%	49	56%	36	42%	0.580 ^2^
	≥0.9	89	51%	39	44%	50	58%	
CAR, median		0.5 (min 0.02–max 6.9)		0.56 (min 0.02–5)		0.5 (min 0.004–max 6.9)		0.489 ^1^
CAR, *n* (%)	<0.5	81	47%	40	45%	41	48%	0.765 ^2^
	≥0.5	93	53%	48	55%	45	52%	
SII, median		595 (min 107–max 4233)		580 (min 107–max 2079)		634 (min 220–max 4233)		0.517 ^1^
SII, *n* (%)	<595	87	50%	47	53%	40	47%	0.363 ^2^
	≥595	87	50%	41	47%	46	53%	
PNI, median		55 (min 39–max 76)		55 (min 42–max 70)		57 (min 39–max 76)		0.068 ^1^
PNI, *n* (%)	<55	64	37%	39	44%	25	29%	0.037 ^2^
	≥55	110	63%	49	56%	61	71%	

^1^ Mann–Whitney U test; ^2^ Chi-squared test; Abbreviations: pCR: Pathological Complete Response; NLR: Neutrophil-to-Lymphocyte Ratio; PLR: Platelet-to-Lymphocyte Ratio; LMR: Lymphocyte-to-Monocyte Ratio; NAR: Neutrophil-to-Albumin Ratio; CAR: C-Reactive Protein to Albumin Ratio; SII: Systemic Inflammation Score; PNI: Prognostic Nutritional Index.

**Table 3:** Univariate and multivariate logistic regression analysis of factors associated with pCR.

Variables		Univariate Analiz		Multivariate Analiz	
		OR (95%CI)	*p*	OR (95%CI)	*p*
Age	<50	1.04 (0.57–1.89)	0.889		
	≥50				
Comorbidity	Absent	1.60 (0.87–2.95)	0.130	1.70 (0.86–3.36)	0.124
	Present				
BMI	<30	0.88 (0.47–1.66)	0.710		
	≥30				
Menopause status	Premenopausal	1.0 (0.55–1.84)	0.980		
	Postmenopausal				
Grade	2 (RC)	1.78 (0.97–3.28)	0.063	1.68 (0.85–3.31)	0.129
	3				
Her2 status	IHC 2+, FISH+	0.24 (0.08–0.68)	0.007	0.23 (0.07–0.71)	0.011
	IHC 3+ (RC)				
T stage	T1–2 (RC)	0.49 (0.23–1.02)	0.058	0.34 (0.14–0.79)	0.012
	T3–4				
N stage	N0–1	0.75 (0.31–1.83)	0.541		
	N2–3				
Stage	2	0.54 (0.28–1.04)	0.067	0.80 (0.26–2.46)	0.709
	3				
Subtype	HR-negative	2.09 (1.11–3.92)	0.021	2.59 (1.25–5.36)	0.01
	HR-positive (RC)				
Ki-67	<30	1.22 (0.66–2.23)	0.519		
	≥30				
Anthracycline-based chemotherapy	Not received	0.87 (0.39–1.92)	0.740		
	Received				
Dual anti-Her2 therapy	Not received (RC)	0.43 (0.21–0.87)	0.02	2.49 (1.16–5.33)	0.019
	Received				
NLR	<2.1 (RC)	1.32 (0.72–2.39)	0.361		
	≥2.1				
PLR	<133 (RC)	1.04 (0.57–1.89)	0.881		
	≥133				
PNI	<55 (RC)	1.94 (1.03–3.63)	0.038	2.89 (1.40–5.99)	0.004
	≥55				
LMR	<4.2 (RC)	1.74 (0.95–3.18)	0.069	1.56 (0.72–3.37)	0.253
	≥4.2				
NAR	<0.9 (RC)	1.19 (0.63–2.28)	0.580		
	≥0.9				
CAR	<0.5 (RC)	0.91 (0.50–1.66)	0.769		
	≥0.5				
SII	<595 (RC)	1.31 (0.72–2.39)	0.363		
	≥595				

Abbreviations: pCR: Pathological Complete Response; BMI: Body Mass Index; IHC: Immunohistochemistry; FISH: Fluorescence In Situ Hybridization; HR: Hormone Receptor; NLR: Neutrophil-to-Lymphocyte Ratio; PLR: Platelet-to-Lymphocyte Ratio; LMR: Lymphocyte-to-Monocyte Ratio; NAR: Neutrophil-to-Albumin Ratio; CAR: C-Reactive Protein to Albumin Ratio; SII: Systemic Inflammation Score; PNI: Prognostic Nutritional Index; RC: Reference Category.

## Data Availability

The data supporting the findings of this study are available from the corresponding author upon reasonable request.

## Supplementary Materials

The following supporting information can be downloaded at: https://www.mdpi.com/article/10.3390/jcm14207271/s1, Table S1: ROC curve analysis of inflammatory and nutritional markers for pCR.

## Abbreviations

The following abbreviations are used in this manuscript:

AUC	Area Under the Curve
BC	Breast Cancer
CAR	C-reactive Protein-to-Albumin Ratio
CI	Confidence Interval
DFS	Disease-Free Survival
ECOG	Eastern Cooperative Oncology Group
ER	Estrogen Receptor
FISH	Fluorescence In Situ Hybridization
HER2	Human Epidermal Growth Factor Receptor 2
IHC	Immunohistochemistry
Ki-67	Ki-67 Proliferation Index
LMR	Lymphocyte-to-Monocyte Ratio
NAR	Neutrophil-to-Albumin Ratio
NAT	Neoadjuvant Therapy
NLR	Neutrophil-to-Lymphocyte Ratio
OR	Odds Ratio
OS	Overall Survival
pCR	Pathological Complete Response
PLR	Platelet-to-Lymphocyte Ratio
PNI	Prognostic Nutritional Index
PR	Progesterone Receptor
RC	Reference Category
ROC	Receiver Operating Characteristic
SII	Systemic Immune-Inflammation Index
