# Supplementary material for: Inflammatory and Nutritional Markers Predicting Pathological Complete Response to Neoadjuvant Therapy in HER2-Positive Breast Cancer: A Multicenter Real-World Study
Source: J Clin Med. 2025 Oct 15;14(20):7271. doi: 10.3390/jcm14207271 (PMC12565550; doi:10.3390/jcm14207271)
Supplement: Supplementary file 1 [file jcm-14-07271-s001.zip › jcm-3874996-supplementary.pdf]

| Table S1: ROC curve analysis of inflammatory and nutritional markers for pCR | | | |
|---|---|---|---|
| ***Variables*** | **AUC** | **95%CI** | **p value** |
| **PNI** | 0.580 | 0.495-0.665 | 0.068 |
| **NLR** | 0.541 | 0.455-0.627 | 0.349 |
| **PLR** | 0.528 | 0.442-0.614 | 0.520 |
| **LMR** | 0.559 | 0.473-0.645 | 0.179 |
| **NAR** | 0.510 | 0.424-0.596 | 0.816 |
| **CAR** | 0.470 | 0.384-0.556 | 0.489 |
| **SII** | 0.528 | 0.442-0.615 | 0.517 |
